# Corrosion Behavior of 700L Automotive Beam Steel in Marine Atmospheric Environment

**DOI:** 10.3390/ma17204964

**Published:** 2024-10-11

**Authors:** Younian He, Yuwei Liu, Chuan Wang, Gongwang Cao, Chunlin He, Zhenyao Wang

**Affiliations:** 1 School of Materials Science and Engineering, University of Science and Technology of China, Shenyang 110016, China; ynhe21b@imr.ac.cn; 2 Institute of Metal Research, Chinese Academy of Sciences, Shenyang 110016, China; ywliu@imr.ac.cn (Y.L.); cwang@imr.ac.cn (C.W.); gwcao@imr.ac.cn (G.C.); 3 Liaoning Provincial Advanced Materials Key Laboratory, Shenyang University, Shenyang 110044, China; ccllhhe@126.com

**Keywords:** high-strength automotive steel, 700L, marine atmospheric corrosion, corrosion depth, rust layer

## Abstract

The marine atmospheric corrosion behavior of 700L high-strength automotive beam steel exposed for 36 months was investigated by scanning electron microscopy, energy dispersive spectroscopy, X-ray diffraction, and electrochemical technology. The corrosion kinetics of 700L steel followed the exponential function: *D* = 4.85*t*^1.23^. The rust layers were mainly composited of γ-FeOOH, α-FeOOH, γ-Fe_2_O_3_, and Fe_3_O_4_, regardless of the exposure duration. With an extended exposure time, the porosity, cracking, and spalling of the rust layers increased, and the densification and thickness uniformity decreased. Electrochemical measurements displayed that the corrosion resistance of the rusted 700L steel gradually decreased with increasing exposure time. A good correlation was found between rust layer composition, microstructure, and corrosion resistance.

## 1. Introduction

700 MPa grade automotive beam steel (700L) is a high-strength micro-alloyed steel developed to meet the needs of lightweight automotive vehicles and energy saving processes, and is widely used in the manufacture of beams and other load-bearing parts of commercial vehicles. As automobile beams are the main components of heavy-duty vehicles, carrying almost all the weight of the goods, the quality of the beams directly impacts the service life and driving safety of the entire vehicle [1]. As we all know, automotive parts are exposed to various outdoor atmospheric environments for a long time, and the mechanical degradation or even failure caused by corrosion is inevitable, so it is especially necessary to examine the corrosion resistance of automotive steels in various atmospheres, especially in highly corrosive marine and industrial atmospheres [2,3,4,5,6].

Atmospheric corrosion of steels is an electrochemical process that occurs under a thin electrolyte layer, which is controlled by temperature, relative humidity, wetting time, electrolyte pH, and contaminants such as chlorides, sulfur dioxide, and nitrogen dioxide [7,8,9,10,11]. Extensive studies have been undertaken on atmospheric corrosion behaviors of metallic materials [12,13,14,15,16,17]. Hou and Liang [13] conducted atmospheric exposure tests on 17 carbon steels and weathering steels in subtropical, temperate, marine, industrial, rural, humid, and dry atmospheres for a period of eight years. The results showed that the humid and hot temperature factors were more important in the long run than the recognized factors such as humidity and SO_2_ and Cl^−^ ion contamination. Corrosion could be very severe if pollution was an additional factor. Liu et al. [12] reported an accelerated corrosion process for both carbon steel Q235 and weathering steel Q450NQR1 in the tropical marine atmosphere of the Spratly Islands, an extreme environment characterized by high temperature, high humidity, high salinity, and high radiation. The corrosivity of this environment is classified as CX [18].

Ma et al. [19] reported that carbon steel Q235 demonstrated a higher corrosion rate in a severe marine atmosphere with a higher Cl^−^ ion deposition rate. The accelerating effects of Cl^−^ ions included promoting the β-FeOOH formation and making the rust layer less compact. However, the influence of lower Cl^−^ ion deposition was mainly to promote the transformation of γ-FeOOH into α-FeOOH. This means that a critical chlorine threshold was required for the β-FeOOH formation. High atmospheric salinity could lead to high β-FeOOH content in mild steel rust layers [20,21,22]. Alcántara et al. [22] conducted a one-year study on mild steel in marine atmospheres with Cl^−^ ion deposition rates of 70–1906 mg·m^−2^·d^−1^, and their experimental results revealed the corrosion was accelerated with increasing Cl^−^ ion deposition rate. With the increase in atmospheric salinity, the rusts exhibited increasingly coarse granulometries, giving rise to less protection and more cracking and detachment. Numerous studies have shown that at atmospheric chloride deposition rates below 600 mg·m^−2^·d^−1^, there appears to be a linear relationship between the corrosion rate of mild steels and the deposition rate of Cl^−^ ions [22,23]. At chloride deposition rates above this value, the corrosion rate appeared to stabilize.

In recent years, there have been an increasing number of reports on the atmospheric corrosion behaviors of high-strength steels [4,24,25,26,27,28]. Yang et al. [4] reported that 1Cr17Ni2 high-strength bolts had higher corrosion susceptibility and loss in tension fatigue life after 36 months of exposure to a marine atmosphere than to an industrial atmosphere. The effects of marine environmental pH [25] and alloying elements [24,27] on the corrosion behaviors of E690, M300, and Q550 high strength steels to marine atmospheric environments have recently been reported in the literature.

To date, studies on the atmospheric corrosion behavior of 700L steel are scarce, which greatly affects its wider application as a high-strength automotive beam steel. In the present study, 700L steel was exposed to the marine atmospheric test station in Wanning, Hainan Province, China. The corrosion kinetics, microstructure, and electrochemical properties of the 700L steel with different exposure times were investigated and the corrosion mechanism was analyzed. This study has a positive effect on the application of 700L steel in harsh atmospheric environments.

## 2. Materials and Methods

### 2.1. Specimen Preparation

The test material (Hebei Iron & Steel Group, Shijiazhuang, China) was high-strength steel 700L with a yield strength of 700 MPa, widely used in the manufacture of automobile beams. The composition of 700L is shown in Table 1. For the outdoor exposure test, 700L steel was processed into 150 mm × 70 mm × 3.5 mm specimens, and the surface was mechanically polished to a surface roughness of 0.8 Ra. Four parallel specimens were used, three of which were used for corrosion weight loss testing and one for rust layer analysis, including rust layer morphology and electrochemical properties. Before outdoor exposure, the specimens were cleaned sequentially with acetone and alcohol, dried, and placed in a desiccator.

### 2.2. Atmospheric Exposure Tests

The exposure test site is located in Wanning City, Hainan Province, China (18°58′ N, 110°05′ E), and is 150 m from the coastline. Its atmospheric environment is typical of the sub-tropical marine climate. The main environment parameters are listed in Table 2, and are characterized by high temperature, high humidity, and high Cl^−^ ion concentration. The surface of the exposure specimens was at an angle of 60° to the horizontal, and the sampling intervals were 6, 12, 24, and 36 months, respectively.

After retrieving the exposed samples, the corrosion products were first scraped off with a razor blade and the collected rust material was used for X-ray diffraction analysis. Then, the rusted specimens were immersed in a room temperature solution containing 3.5 g C_6_H_12_N_4_, 500 mL HCl (ρ = 1.19 g/mL) (Sinopharm Reagent, Shanghai, China), and 500 mL distilled water. The immersion time was determined by the rusting degree of the specimens, during which the residual rust on the specimens was scrubbed with a brush until it was completely removed. The specimens were washed with distilled water and ethanol after descaling, dried, stored in a desiccator for 24 h, and then weighed with an accuracy of 0.1 mg.

### 2.3. Corrosion Product Analysis

The macroscopic morphology of the 700L specimens was observed with a Nikon digital camera (D50), and the micro-morphology and composition of the rust layer was analyzed with an FEI Inspect F50 scanning electron microscope (SEM, FEI, OR, USA) and an accompanying energy-dispersive spectrometer (EDS) with accelerating voltages ranging from 15–25 kV. A gold coating was sputtered onto the surface of the specimens prior to the SEM/EDS analysis.

The corrosion products scraped with a razor blade were ground into powders and then analyzed using a Rigaku-D/max 2500PC X-ray diffractometer (XRD, Rigaku, Tokyo, Japan) with a Cu target at a voltage and current of 50 kV and 3000 mA, respectively. The scanning angle ranged from 10° to 80° at a scanning rate of 10°/min and a scanning step size of 0.02°.

### 2.4. Electrochemical Measurements

The electrochemical behavior of the rusted 700L steels for different exposure times was measured on a PARSTAT 2273 potentiostat/galvanostat (Princeton, NJ, USA). The measurements were performed by a three electrode system with the rusted steel as working electrode, the saturated calomel electrode (SCE) as reference electrode, and a graphite rod as auxiliary electrode. The electrolyte was a 0.1 mol/L Na_2_SO_4_ aqueous solution, used due to its stability and minimal effect on the rust layer. Open circuit potential (OCP) vs. time curve was measured until the OCP reached stability. An electrochemical impedance spectroscopy (EIS) measurement was made at a frequency range of 10^5^ Hz to 10 mHz with an AC excitation signal amplitude of 10 mV, and the EIS data were analyzed by ZSimpWin software 3.3. Potentiodynamic polarization was conducted at a scan rate of 0.333 mV/s.

## 3. Results and Discussion

### 3.1. Corrosion Kinetics

Generally, the corrosion rate of a metallic material in an outdoor atmospheric environment is expressed in terms of the corrosion depth, *D*, by the following Equation (1):

*D* = 10,000 (*W*_0_ − *W*_t_)/*ρS* (1)

where *D* is the corrosion depth (μm), *W*_0_ and *W*_t_ are the weight (g) of the sample before exposure and after descaling, respectively, *ρ* is the density of 700L steel, equal to 7.86 g/cm^3^, and *S* is the surface area of the exposed specimen (cm^2^).

**Figure 1:**
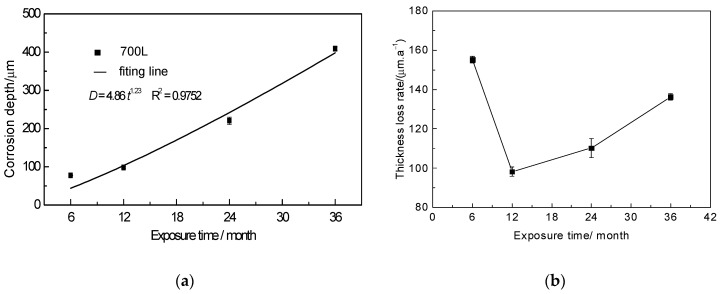
Variations of corrosion depth (**a**) and corrosion rate (**b**) of 700L steel with exposure time.

Figure 1a shows the variation curve of *D* with time for 700L steel exposed to the atmosphere. As can be seen, the *D* value increases gradually with the increase of exposure time. Within 6 months, the *D* value increases rapidly to 77.59 ± 0.87 μm, indicating that the corrosion products (i.e., rust layer) have a limited role in hindering the corrosion. When the exposure time increases to 12 months, *D* is 98.23 ± 2.27 μm, and the corrosion rate slows down obviously, revealing that the corrosion products at this stage have an obvious effect on slowing down the corrosion. However, when the exposure time is extended to 24 months and 36 months, the corresponding *D* values are 220.55 ± 9.68 μm and 408.86 ± 4.94 μm, respectively, and the corrosion rate begins to accelerate significantly, revealing that the rusts formed by extending the exposure time have weakened the hindering role on the corrosion process, but it is still stronger than that of the rust layer formed before 6 months.

Generally, the corrosion depth of steels exposed to marine atmospheric environment follows the well-known exponential Equation (2):

*D* = *At*^n^ (2)

where *D* is the corrosion depth (μm), *t* is the exposure time (months), and *A* and *n* are constants. The larger the *A* value, the higher the steel corrosion rate in the first month. The *n* value reflects the protective performance of the rust layer; the larger the value of *n*, the worse the protective performance. The actual exposure data and the regressed curve are presented in Figure 1a. According to the regressed results, *D* = 4.86*t*^1.23^ and the correlation coefficient of the regressed equation is *R*^2^ = 0.9752. It has been found that the *n*-values of various steels exposed to most atmospheric environments are not greater than 1 [13,17], and that only in a few environments does *n* > 1 occur, e.g., the *n*-values of various carbon steels exposed to the same locations as in the present study range from 1.22 to 1.89 [13]. For high-strength steels, such as the 921A steel exposed to the marine atmospheres in Xiamen and Qingdao, China, the *n*-values are only 0.37 and 0.67, respectively [26]. The *n*-value of 700L steel is 1.23, which shows that the rust layer does not have a significant protective effect, with the corrosion progress exhibiting an accelerated trend owing to *n* > 1.

Figure 1b shows the variation curves of corrosion rate *V* of the samples with different exposure times. As seen from the figure, the *V* value is 155.17 ± 1.75 μm/a for 6 months, and when the exposure time is extended to 12 months, the *V* value rapidly decreases to a minimum of 98.22 ± 2.27 μm/a. Continuing to extend the exposure time, the *V* value begins to gradually increase again, and it is 136.29 ± 1.65 μm/a for 36 months. This reveals that at the early stage of exposure (less than 6 months), a strong protective rust layer is quickly formed on the 700L, resulting in reduced corrosion rate between 6 and 12 months. However, when the exposure time exceeds 12 months, the protective property of the rust layer is gradually lost with increasing exposure time, leading to accelerated corrosion. This is similar to the results of marine atmospheric corrosion tests on Q235 steel [29,30].

### 3.2. Macroscopic Corrosion Product Morphology

**Figure 2:**
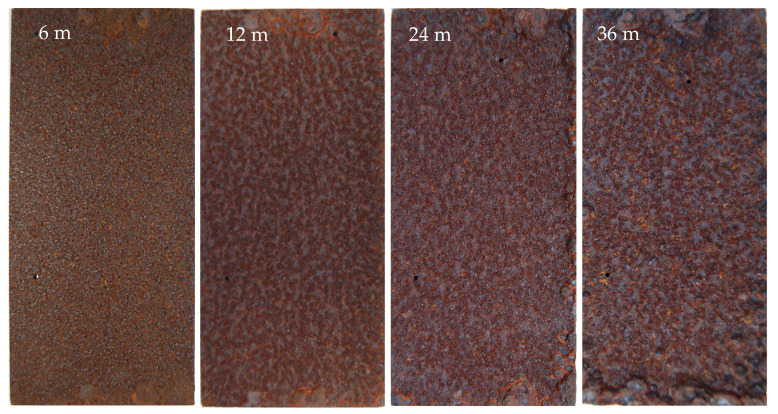
Evolution of the macro-morphologies of the rust layers on 700L steel with different exposure times.

Figure 2 shows the macroscopic morphologies of the rust layers on the 700L steel with various exposure times. After 6–36 months of exposure in marine atmospheric environment, the specimen surfaces are covered with a thick rust layer. The 6-month rust layer is predominantly reddish-brown, turning dark brown and dotted with reddish-brown and orange-brown spots as the exposure time increases [31]. Distinguished by color, the reddish-brown rust is mainly γ-Fe_2_O_3_, whereas the dark brown rust is primarily α-FeOOH [32]. Moreover, the 6-month rust layer is relatively smooth and compact, but as the exposure time increases, the corrosion gradually intensifies, and the surface of the rust layer becomes rougher and more uneven, and the rust layer is locally peeled off.

### 3.3. Microscopic Corrosion Product Morphology

#### 3.3.1. Surface Morphology

**Figure 3:**
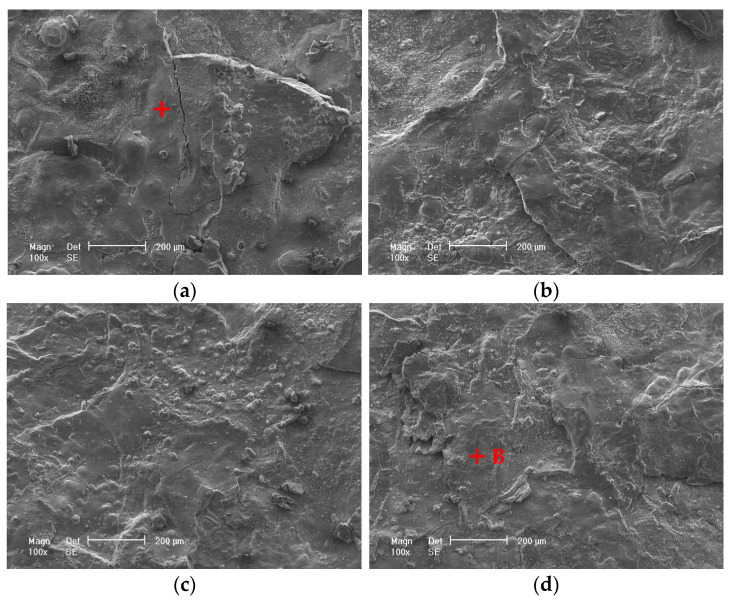
Surface morphologies of the rust layers for various exposure times. (**a**) 6 months, (**b**) 12 months, (**c**) 24 months, (**d**) 36 months.

**Figure 4:**
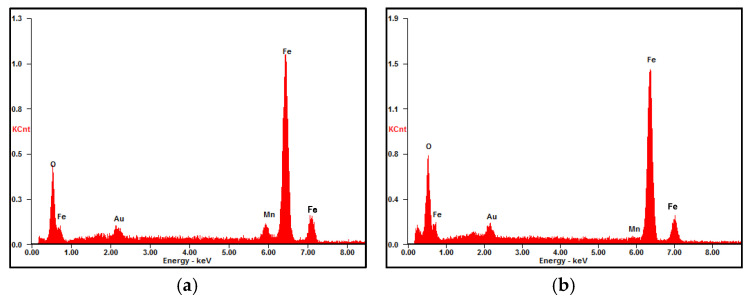
Compositions of points marked in the rust layers in Figure 3a,d. (**a**) point A in Figure 3a, (**b**) point B in Figure 3d.

Figure 3 displays the microscopic morphologies of the rust layers after different exposure times. The rust layer is very thick with some cracks and localized flaking. The surface of the 6-month exposure is relatively compact and smooth. In contrast, the surfaces of the 12-, 24-, and 36-month rust layers become a little rough, with many round particles distributed on the surfaces, which should be α-FeOOH. Similar morphologies have been reported by Ma et al. [19] and Oh et al. [33]. Figure 4 presents the rust compositions obtained by EDS analysis on points A and B shown in Figure 3a,d. The compositions of the rust layers at 6 and 36 months are basically the same, containing mainly O, Fe, and a small amount of Mn, i.e., the rust layers consist of oxides of Fe and Mn. No Cl is found in the rust layer, probably due to the fact that the rainwater washed away the chlorine salt deposits on the surface during the exposure period [23,29,34]. In addition, a small amount of gold appeared in Figure 4, which is the result of gold spraying on the rust layer to improve its electrical conductivity before SEM observation.

#### 3.3.2. Cross-Sectional Morphology

**Figure 5:**
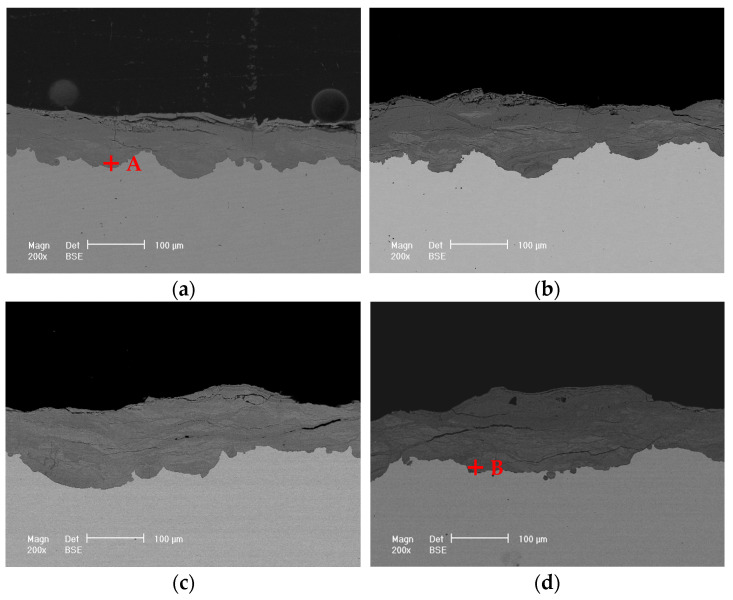
Cross-sectional morphologies of the rust layers changed with exposure times. (**a**) 6 months, (**b**) 12 months, (**c**) 24 months, (**d**) 36 months.

In natural environments, the rust layer formed on the steel surface is usually uneven and heavily cracked. When exposed outdoors, the loose portion of the outer layer of the rust layer is easily stripped off due to rainfall, outdoor sampling, or SEM sample preparation, resulting in the actual rust layer analyzed being the relatively dense portion that is retained. Figure 5 presents the cross-sectional images of the rust layers. It can be clearly seen that after exposure for different lengths of time, (1) the thickness of the rust layer on 700L steel is obviously uneven, with localized areas being thicker; (2) A large number of cracks occur in the rust layer, in which the transverse cracks are more, wider, and longer, while the longitudinal cracks are relatively fewer, narrower, and shorter; (3) The rust layer close to the steel substrate is relatively dense, whereas the outer layer is more loose and porous; (4) The longer the exposure time, the more serious the flaking of the rust layer, resulting in an uneven surface. Especially after 24 and 36 months of exposure, the outer rust layer is peeling off severely in some places, leaving only a very thin layer of relatively dense rust. It is believed that the local detachment is associated with the distinct difference in molar volume of the rust phases [35].

**Figure 6:**
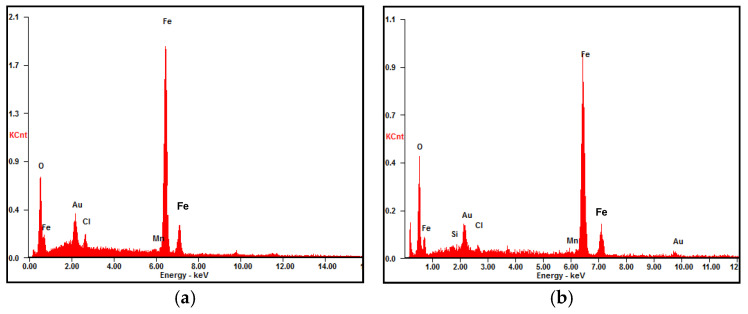
Rust compositions of points marked in Figure 5a,d. (**a**) point A in Figure 5a, (**b**) point B in Figure 5d.

The compositions of points A and B on the rust layers in Figure 5 analyzed by EDS are presented in Figure 6. A small amount of Cl is detected from the bottom of the rust layer. Similar results had been reported by others [12,22]. Cl seemingly comes from chlorides in the marine atmosphere, which penetrates through cracks or pores in the rust layer. There is no doubt that the presence of Cl^−^ ions accelerates the corrosion process of the steel substrate.

### 3.4. Corrosion Morphology

**Figure 7:**
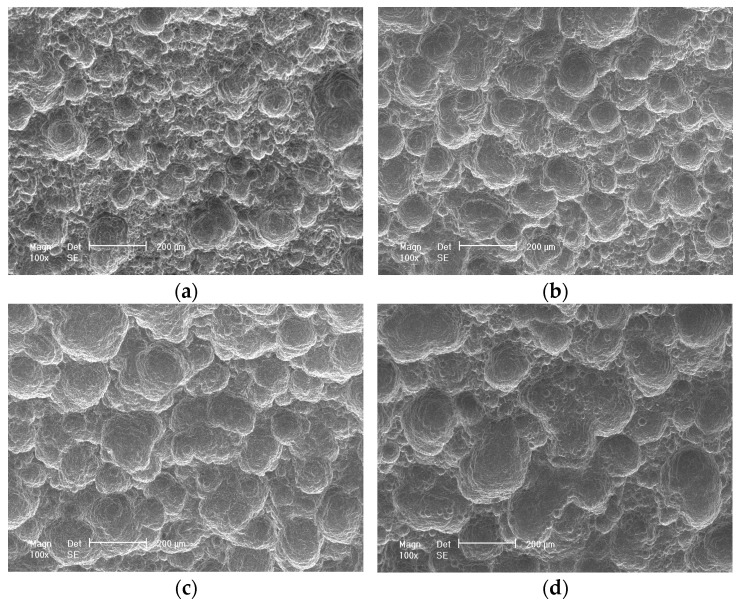
Corrosion morphologies of 700L steel exposed for different times. (**a**) 6 months; (**b**) 12 months; (**c**) 24 months; (**d**) 36 months.

Figure 7 presents the corrosion morphologies of 700L steel after 6–36 months of exposure and removal of rust layers. The 700L steel is subjected to homogeneous corrosion in the marine atmospheric environment, with a large number of pits distributed on the steel surface, similar to the craters on the surface of the Moon. Most of the pits are nearly circular and their diameter increases with the exposure times. After 6 months of exposure, the sizes of the pits on the 700L steel surface are not uniform, with some pits becoming larger and others significantly smaller. However, after 12 months of exposure, the pits are approximately the same size and cover almost the entire surface. After 24 months of exposure, the round pits gradually interconnect with each other and evolve into larger pits with relatively flat bottom but irregular shape, with many smaller pits appearing at the bottom. It is believed that the appearance of small pits is related to inclusions in the steel. With the increase in exposure time, the small pits gradually grow through successive mergers to become isolated large pits (Figure 7a,b). Similarly, large pits grow and merge, evolving into larger irregular pits with relatively flat bottoms (Figure 7c,d). The above processes are repeated over and over again, resulting in the formation of the characteristic morphology in marine atmospheric environment.

### 3.5. Phase Composition of the Rust Layer

**Figure 8:**
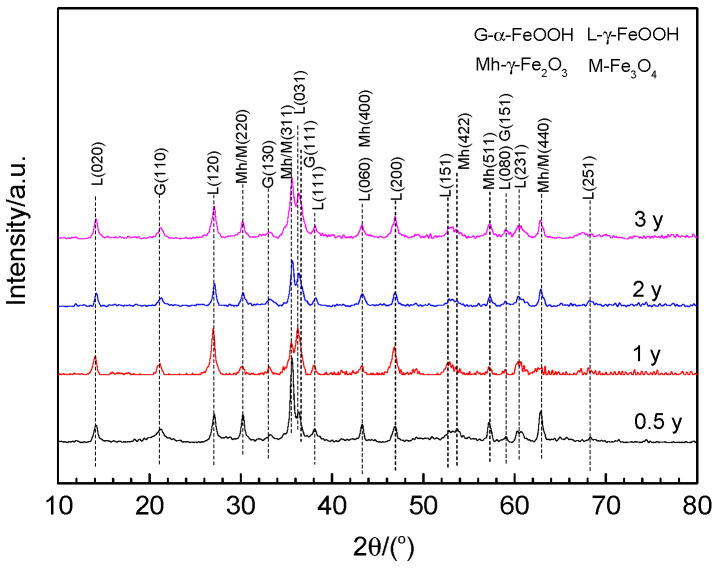
XRD patterns of the rusts on 700L steel for different exposure times.

The rust powders scraped from the rust layers after different exposure times were analyzed by XRD and the relative results are presented in Figure 8. Clearly, the rust phase compositions are exactly the same for the 700L steel exposed to the South China Sea atmosphere, mainly γ-FeOOH (lepidocrocite; JCPDF#08-0098), α-FeOOH (goethite; JCPDF#29-0713), γ-Fe_2_O_3_ (maghemite; JCPDF#39-1346), and Fe_3_O_4_ (magnetite; JCPDF#19-0629), independent of the exposure time. Apparently, the diffraction peaks of γ-FeOOH are stronger than those of α-FeOOH, which is consistent with the corrosion layer compositions of carbon steel at Wanning exposure station [19] and in other marine atmospheric environments [33]. The diffraction peaks of Fe_3_O_4_ and γ-Fe_2_O_3_ are also relatively high, especially for the rust layer exposed for 6 months. Among them, the peak intensities of γ-Fe_2_O_3_ are remarkably higher than those of Fe_3_O_4_. This is in agreement with the rust layer compositions analyzed by using Mössbauer and Raman spectra [33] and by XRD [25]. Both maghemite and magnetite phases belong to the spinel structure, which could be the main rust constituents in severe marine atmospheres [36]. Alcántara et al. [22] found the spinel phase content rose at the expense of the lepidocrocite content in a severe aggressive marine atmosphere. In general, β-FeOOH (akaganeite) is also found in the rust layer on carbon steel exposed to marine atmospheres with high chloride concentration [37]. There do not appear to be β-FeOOH diffraction peaks in Figure 8, perhaps due to the fact that the present test site is relatively far away from the coastline and the atmospheric deposition of Cl^−^ ions is relatively low [19], or because the β-FeOOH content in the rust layer is so small that it cannot be detected by XRD. In fact, β-FeOOH is an unstable phase that can deteriorate the marine atmospheric corrosion resistance of mild steels, thus accelerating the corrosion process.

The rusting involves a series of chemical and electrochemical reactions. When 700L steel is exposed to a marine atmosphere, the steel surface will be covered with a thin electrolyte layer, then the electrochemical corrosion process is initiated. Fe is quickly oxidized to Fe^2+^ [38] and converted to Fe(OH)_2_ or FeOH^+^ by hydroxylation. The FeOH^+^ complexes are thermally unstable and easily transformed to γ-FeOOH by oxidation of FeOH^+^ with O_2_ dissolved in the electrolyte layer [39]. The precipitation and crystallization of γ-FeOOH is accelerated in fine weather through drying. During the dry/wet cycle process, γ-FeOOH can partially be changed into amorphous ferric oxyhydroxide, and then converted to α-FeOOH by solid state transformation. In addition, a part of γ-FeOOH can be transformed into Fe_3_O_4_, which can be further oxidized to γ-Fe_2_O_3_. The main reactions that occur during rusting are as follows [12,32,39]:

Fe → Fe^2+^ + 2e^−^ (3)

Fe^2+^ + H_2_O → Fe(OH)^+^ + H^+^ (4)

2Fe(OH)^+^ + O_2_ + 2e^−^ → 2γ-FeOOH (5)

γ-FeOOH → Fe_x_(OH)_3−2x_ → α-FeOOH (6)

8γ-FeOOH + Fe^2+^ + 2e^−^ → 3Fe_3_O_4_ + 4H_2_O (7)

4Fe_3_O_4_ + O_2_ → 6γ-Fe_2_O_3_ (8)

### 3.6. Electrochemical Corrosion Properties

**Figure 9:**
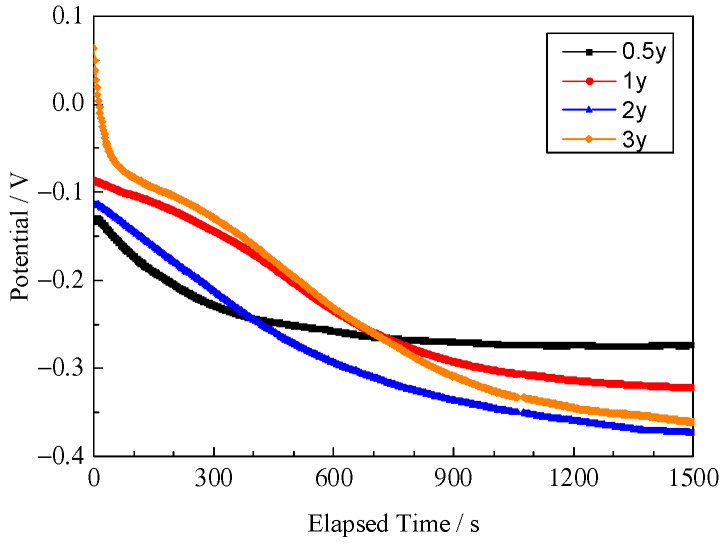
Changes of OCP of the rusted steels with exposure times.

To further understand the electrochemical property of the rusted 700L steel, the OCP was measured in 0.1 mol/L Na_2_SO_4_ aqueous solution. Figure 9 presents the variation curves of the OCP as a function of the immersion time. The OCP of the specimens exposed in an outdoor marine atmosphere for 6 months reaches stability after 400 s, whereas for the 700L steel specimens exposed for 12–36 months, their OCP reaches stability after up to 1000 s. The stable OCP values of the rust layers for 6, 12, 24, and 36 months are −0.274 V, −0.322 V, −0.374 V, and −0.361 V, respectively. The OCP values gradually shift negatively as the exposure time increases, indicating that the inertness of the rust layers become weaker, i.e., the protection becomes worse. The OCP values of the 24- and 36-month rust layers differ only by ~0.013 V, exhibiting similar inertness of the two rust layers. The OCP results are consistent with the SEM morphology shown in Figure 5.

**Figure 10:**
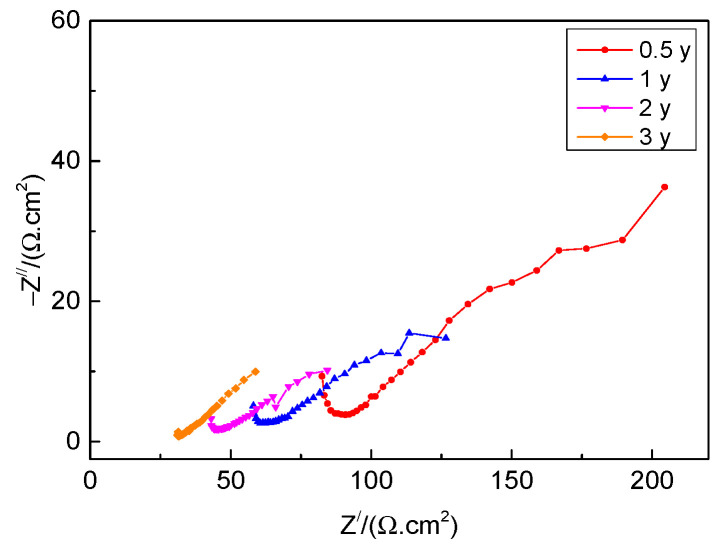
Nyquist plots of the rusted 700L steel for different exposure times.

**Figure 11:**
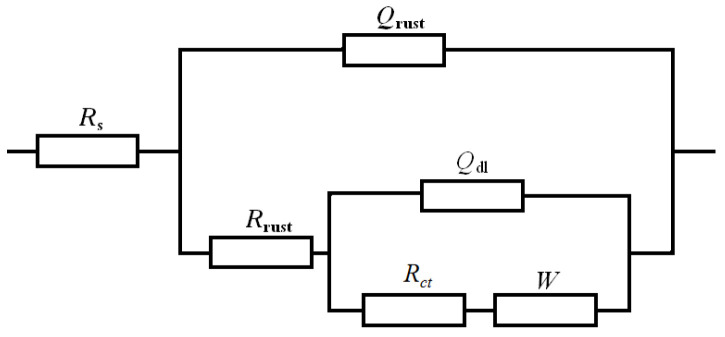
Equivalent circuit for fitting the EIS of the rusted 700L steel.

EIS measurements of the rusted 700L steels for various exposure times were conducted in Na_2_SO_4_ solution. Figure 10 shows the EIS data with Nyquist plots. This is a heavily compressed EIS plot with significantly higher impedance in the real section than in the imaginary section. Such EIS plots are similar to the shape of the EIS of mild steel Q235 exposed to the marine and industrial atmospheric environments [19,40]. With the increase of exposure time, the EIS curve shifts to the left and the impedance modulus |*Z*| decreases gradually, showing that the corrosion resistance of the rust layer decreases. The EIS data can be fitted by the *R*(*Q*(*R*(*Q*(*RW*)))) equivalent circuit in Figure 11. Where *R*_s_ is the solution resistance, *R*_rust_ and *Q*_rust_ stand for the resistance and constant phase element of the rust layer, respectively, *R*_ct_ and *Q*_dl_ stand for the charge transfer resistance and constant phase element of the double layer, respectively, and *W* represents Warburg impedance.

**Figure 12:**
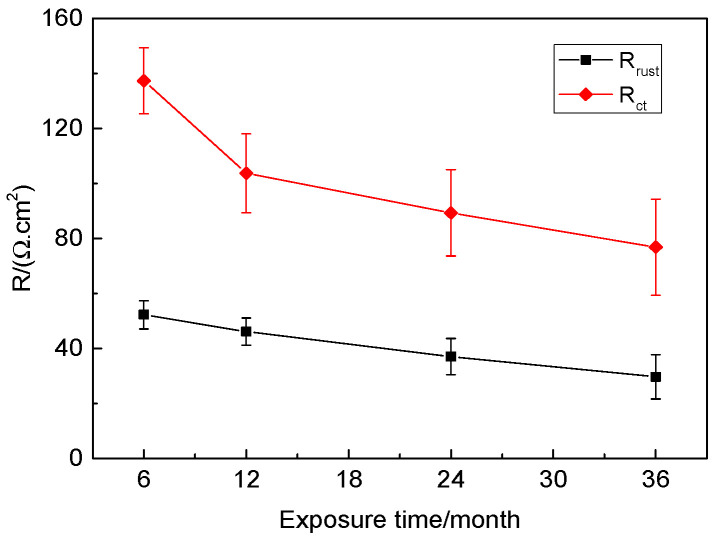
*R*_rust_ and *R*_ct_ vs. exposure times of the rusted 700L steel.

The fitted EIS parameters are presented in Table 3. It is clearly shown that as the exposure time is prolonged from 6 months to 36 months, *R*_rust_ and *R*_ct_ values decrease gradually from 52.27 Ω·cm^2^ to 29.66 Ω·cm^2^ and from 137.31 Ω·cm^2^ to 76.83 Ω·cm^2^, respectively, and the values of *Q*_rust_ and *Q*_dl_ gradually increase, showing that the protective properties of the rust layer decreases, thus accelerating the corrosion progress of the steel substrate [40]. The variation curves of *R*_rust_ and *R*_ct_ with exposure times are presented in Figure 12, and they exhibit nearly linear downward trends. Generally, the larger the *R*_rust_ value, the better the protective properties of the rust layer, and the bigger the *R*_ct_ value, the smaller the corrosion rate of the steel substrate. The EIS results are in agreement with the OCP (Figure 9) results.

**Figure 13:**
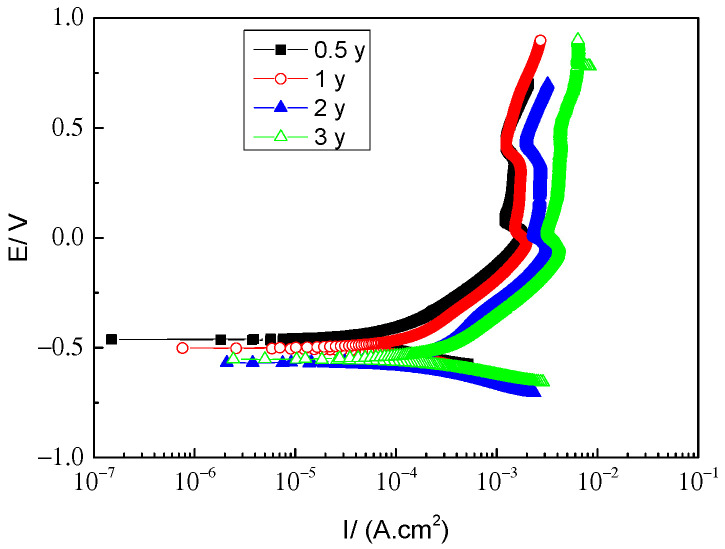
Potentiodynamic polarization curves of 700L steel after various exposure times.

Figure 13 presents the variation of the potentiodynamic polarization curves of the rusted 700L steels for different exposure times in an 0.1 mol/L Na_2_SO_4_ aqueous solution. Clearly, all the polarization curves have similar shapes, indicating similar polarization behavior and corrosion mechanism. Table 4 lists the values of the corrosion current density, *I*_corr_, and corrosion potential, *E*_corr_, obtained by Tafel fitting the polarization curves in Figure 13. *E*_corr_ shifts negatively from −0.460 V to −0.568 V as the exposure time is prolonged from 6 months to 24 months. After that, *E*_corr_ value is almost unchanged. However, *I*_corr_ gradually increases from 4.340 × 10^−5^ A·cm^−2^ to 2.863 × 10^−4^ A∙cm^−2^ with increasing exposure time from 6 months to 36 months. Similarly, the polarization resistance *R*_p_ gradually decreases from 604.0 Ω·cm^2^ to 130.4 Ω·cm^2^ with increasing exposure time. Generally, the more positive the *E*_corr_, the smaller the *I*_corr_ or the larger the *R*_p_, the better the corrosion resistance of the rusted 700L steel. As a result, the protective properties of the rust layer formed on the surface of 700L steel gradually deteriorate when increasing exposure time. The polarization results are consistent with those of the OCP (Figure 9) and EIS (Figure 10) tests. Based on the above electrochemical measurements, it can be reasonably deduced that the optimum protective performance of the rust layer occurred before 6 months. Therefore, it is of great significance to conduct research on protective coatings or treatments in order to improve the long-term corrosion resistance of 700L steel in harsh marine atmospheres. Related research work has been conducted in our group.

The protective property of the rust layer is closely related to its structure and composition [41]. Usually, a dense rust layer has better protective properties. The presence of pores and cracks in the rust layer, especially those that pass directly through the layer to the substrate, can greatly reduce the corrosion resistance of the rust layer. Pores and cracks can easily become a direct path for aggressive Cl^−^ ions and O_2_ molecules to reach the substrate, leading to accelerated corrosion of the metal. The thickness and composition of the rust layer also have a certain impact on the corrosion resistance. Generally, a thicker rust layer exhibits better corrosion resistance. Since flaking usually results in a significantly non-uniform thickness of the rust layer, the thinner portions are often the weakest points, favoring the entry of corrosive media. The higher the contents of α-FeOOH and Fe_3_O_4_/γ-Fe_2_O_3_, or the lower the contents of β- and γ-FeOOH in the rust layer, the better the protection, and vice versa [23,41]. The SEM morphology revealed cracks, pores, flaking, and uneven thickness (Figure 3 and Figure 5), and the XRD patterns showed the presence of unstable γ-FeOOH in the rust layers, all of which could do great damage to the corrosion resistance of the rusted steel.

It is worth noting that the electrochemical results show that the steel rusted for 6 months has the best corrosion resistance, while the corrosion rate based on corrosion weight loss is the largest (Figure 1). The main reason for this is that the former reflects the corrosion properties of the rust layer solely at the 6-month point in time, while the latter is the average corrosion performance over the entire 6-month period. When 700L steel is exposed to the marine atmosphere over a relatively short period of time (less than 6 months), a structurally dense rust layer is formed with virtually no cracks or pores, exhibiting significant protective properties and greatly reducing the subsequent corrosion rate. This protective effect persists beyond 6 months due to the relatively dense structure of the 6-month rust layer (Figure 3 and Figure 5) and the relatively strong Fe_3_O_4_/γ-Fe_2_O_3_ diffraction peaks (Figure 8). Consequently, the corrosion rate of the rust layer at the 6th month should be very small, verified by the electrochemical measurements with the strongest corrosion resistance of the rust layer at the 6th month. However, the high corrosion rate at the beginning of the 700L exposure results in a higher average corrosion rate based on the corrosion weight loss over the first 6 months. The increase in cracking, porosity, and rust flaking and the decrease in the content of stabilizing components (e.g., Fe_3_O_4_/γ-Fe_2_O_3_ phase) over the 6 to 12 month exposure period means that the rust layer of the 12th month has lost its protective properties, resulting in an increase in the corrosion rate of the rust layer as evidenced by the electrochemical tests, even though the average rate of thickness loss, as measured by the weight loss, is the lowest. Similarly, the corrosion resistances of the layers continue to decrease at months 24 and 36.

## 4. Conclusions

(1) The corrosion kinetics of 700L steel exposed to the marine atmosphere for 36 months follow the exponential function *D* = 4.85*t*^1.23^, and the corrosion process is accelerated with exposure time.
(2) The rust phases on the 700L steel are mainly composited of γ-FeOOH, α-FeOOH, Fe_3_O_4_, and γ-Fe_2_O_3_, regardless of the exposure duration.
(3) Lots of porosity, spalling, and cracks in the rust layer are present, which are the easy pathways for chloride ions and oxygen to reach the steel substrate, thereby accelerating the corrosion process.
(4) Based on the electrochemical data from the OCP, polarization curves, and EIS, the corrosion resistance of rusted 700L steel decreases with exposure time, which is associated with the increase in porosity, cracking, and spalling.

## Figures and Tables

**Table 1:** The composition of 700L steel used (wt %).

C	Mn	S	P	Si	Als	Cu	Cr	Ni	Fe
0.072	1.68	0.0041	0.013	0.132	0.030	0.016	0.028	0.022	Bal.

**Table 2:** The environment parameters of the exposure location.

Average Temperature (°C/year)	Average Relative Humidity (%)	Average Sunshine Time (h/year)	Average Rain Time (h/year)	Average Cl^−^ Ions Deposition (mg·m^−2^·d^−1^)
24.6	86	2043	391.7	50.39

**Table 3:** Electrochemical parameters fitted from the EIS in Figure 10.

Time/ Year	*R_s_*/ (Ω·cm^2^)	*Q*_rust_/ (Ω^−1^·cm^−2^·s^−n^)	*n*_rust_	*R*_rust_/ (Ω·cm^2^)	*Q*_dl_/ (Ω^−1^·cm^−2^·s^−n^)	*n*_dl_	*R*_ct_/ (Ω·cm^2^)	*W*/ (Ω^−1^·cm^−2^·s^−0.5^)
0.5	6.43 × 10^−5^	1.344 × 10^−9^	0.9958	52.27	0.007753	0.09241	137.31	0.003124
1	9.999 × 10^−4^	1.563 × 10^−9^	1	46.12	0.01663	0.1126	103.7	0.004857
2	9.993 × 10^−4^	1.852 × 10^−9^	1	37.03	0.02868	0.1281	89.30	0.009986
3	0.0989	2.001 × 10^−9^	0.9901	29.66	0.05399	0.2024	76.83	0.01298

**Table 4:** Electrochemical parameters calculated from the polarization curves in Figure 13.

Time/year	*I*_corr_/(A·cm^−2^)	*E*_corr_/V	*β*_c_/(V·dec^−1^)	*β*_a_/(V·dec^−1^)	*R*_p_/(Ω·cm^2^)
0.5	4.340 × 10^−5^	−0.460	0.103	0.144	604.0
1	1.166 × 10^−4^	−0.502	0.142	0.314	364.6
2	2.346 × 10^−4^	−0.568	0.138	0.436	194.3
3	2.863 × 10^−4^	−0.551	0.112	0.368	130.4

## Data Availability

The original contributions presented in the study are included in the article, further inquiries can be directed to the corresponding author.
